# Supplementary material for: Assessment of transcriptomic constraint-based methods for central carbon flux inference
Source: PLoS One. 2020 Sep 9;15(9):e0238689. doi: 10.1371/journal.pone.0238689 (PMC7480874; doi:10.1371/journal.pone.0238689)
Supplement: S3 Dataset — The genome-scale metabolic model of PCC 6803 developed by Knoop et al. [24]. Individual models in SBML format (.xml) with set constraints used AC, DC, etc. are included. Transcriptomic data (.csv) from by Dr. Le You (University of California San Diego, USA) and Dr. Yinjie Tang (Washington University in St. Louis, USA) [12]. Predicted fluxes generated using these data. MATLAB scripts used for calculating correlations (.m). (GZ) [file pone.0238689.s003.gz › PCC6803_model_and_data/models/README.rtf]

pcc6803 README:Transcriptomic methods (E-Flux2 and SPOT) were run using models that did not directly include uptake rates (unconstrained 1000 flux instead).Use AC_* models for the DC correlation fluxes in Figure 3A.Use DC* to generate just the pFBA_mf black bars in Figure 3A.Use AC_mixo as the model and the corresponding gene expression set, to generate the “AC mixotrophic” plot for both transcriptomic (and non-transcriptomic pFBA (blue) bars) correlations in Figure 3B.
