# Supplementary material for: Assessment of transcriptomic constraint-based methods for central carbon flux inference
Source: PLoS One. 2020 Sep 9;15(9):e0238689. doi: 10.1371/journal.pone.0238689 (PMC7480874; doi:10.1371/journal.pone.0238689)
Supplement: S4 Dataset — The genome-scale metabolic model of PCC 7002 from Qian et al. [26]. Individual models in SBML format (.xml) with set constraints used AC, DC, etc. are included. Transcriptomic data (.csv) from Ludwig and Bryant [25]. Predicted fluxes generated using these data. MATLAB scripts used for calculating correlations (.m). (GZ) [file pone.0238689.s004.gz › PCC7002_model_and_data/models/README.rtf]

pcc7002 README:DC* models were used to make Figure 4A. DC implies constrained carbon source (CO2).AC* models were used to make FIg. 4B.
