# Supplementary material for: Assessment of transcriptomic constraint-based methods for central carbon flux inference
Source: PLoS One. 2020 Sep 9;15(9):e0238689. doi: 10.1371/journal.pone.0238689 (PMC7480874; doi:10.1371/journal.pone.0238689)
Supplement: S5 Dataset — Python code used in plotting and analysis (.ipynb), and tab-delimited tables of correlations generated. See README for details. (GZ) [file pone.0238689.s005.gz › plotting_scripts_and_correl_tables/README.rtf]

plotting README:All .ipynb files were run in jupyter lab.The scripts and corresponding files referred within the scripts are all in this directory. No additional pathing needed.Some file naming schemes may have been replaced (e.g 8C refers to AC, trueAC refers to FullAC). 
