# Supplementary material for: Assessment of transcriptomic constraint-based methods for central carbon flux inference
Source: PLoS One. 2020 Sep 9;15(9):e0238689. doi: 10.1371/journal.pone.0238689 (PMC7480874; doi:10.1371/journal.pone.0238689)
Supplement: S1 File — Supporting figures (S1–S8 Figs) and tables (S1 and S2 Tables) with associated captions. (PDF) [file pone.0238689.s006.pdf]

Supplementary Information

Assessment of transcriptomic constraint-based methods for central carbon flux inference

Siddharth Bhadra-Lobo[1,], Min Kyung Kim[1], and Desmond S. Lun[1,2,3]

[1]Center for Computational and Integrative Biology, Rutgers, The State University of New Jersey, Camden, NJ, USA
[2]Department of Computer Science, Rutgers, The State University of New Jersey, Camden, NJ, USA
[3]Department of Plant Biology, Rutgers, The State University of New Jersey, New Brunswick, NJ, USA

S1 Fig: *B. subtilis* carbon source clustering ....

S2 Fig: PCC 6803 pFBA_mf vs pFBA ....

S3 Fig: *E. coli* flux directionality AC vs Full AC low correlation carbon sources..................................

S4 Fig: *E. coli* flux directionality AC vs Full AC all carbon sources ....

S5 Fig: *B. subtilis* flux directionality AC vs Full AC all carbon sources ....

S6 Fig: PCC 6803 flux directionality DC and AC conditions ....

S7 Fig: PCC 7002 flux directionality DC and AC conditions ....

S8 Fig: Spearman correlations equivalents of main figures 1-4 ....

S1 Table. Measured uptake rates ....

S2 Table. PCC 6803 Pentose Phosphate Pathway Fluxes..

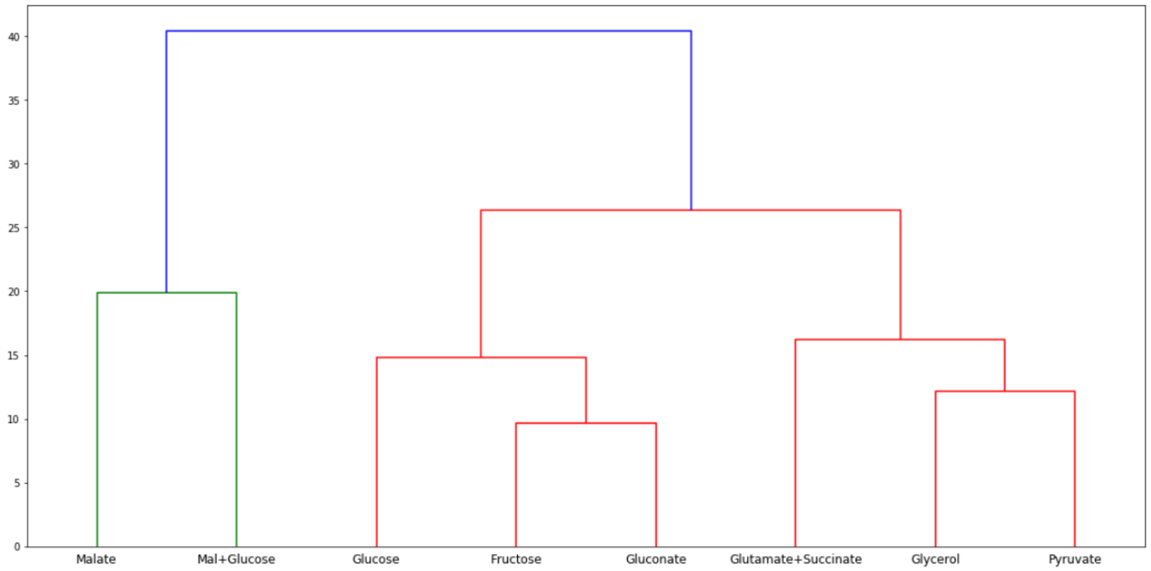

S1 FigA. *B. subtilis* carbon source clustering: *B. subitilis* single and double (Malate+Glucose and Glutamate+Succinate) carbon source measured flux data dendogram. Ward linkage was used and minimizes the sum of squared differences within all clusters based on measured flux values.

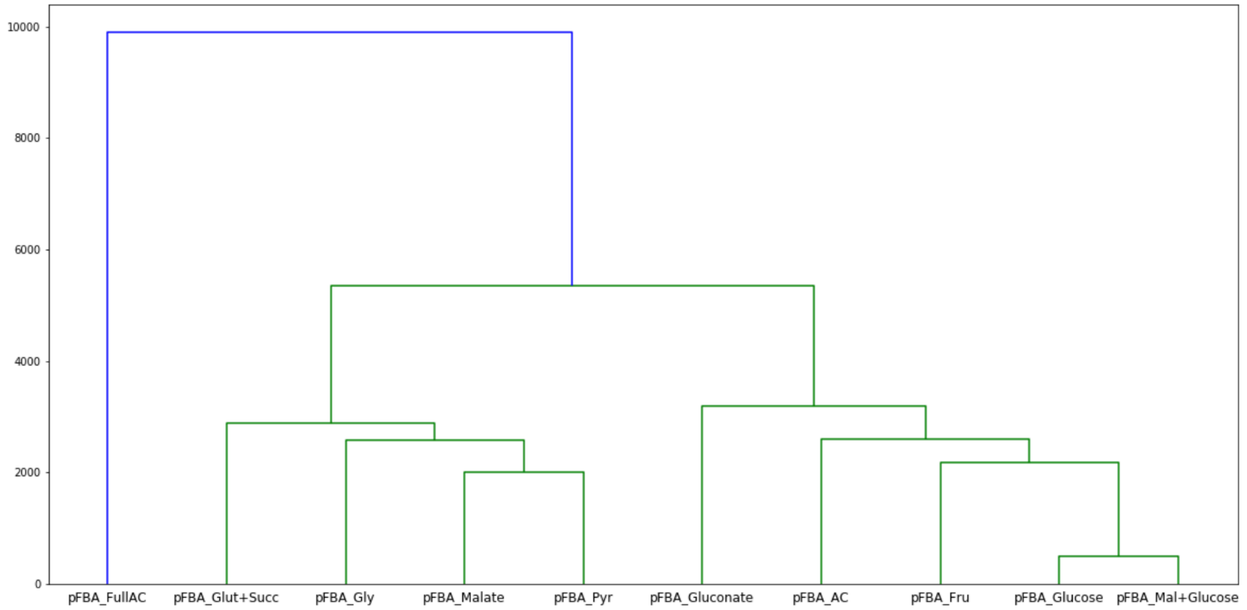

S1 Fig B. *B. subtilis* pFBA carbon source based flux distribution clustering: All *B. subitilis* pFBA flux distributions. AC refers to the single flux pattern produced by pFBA when allowed all 8 carbon sources. FullAC refers to the single pFBA flux distribution produced under all possible carbon sources available in the model. FullAC distribution is the outgroup. Glut+succ looks similar to the other TCA metabolite fluxes, while the AC distribution looks similar to the the sugars in the right subclusters.

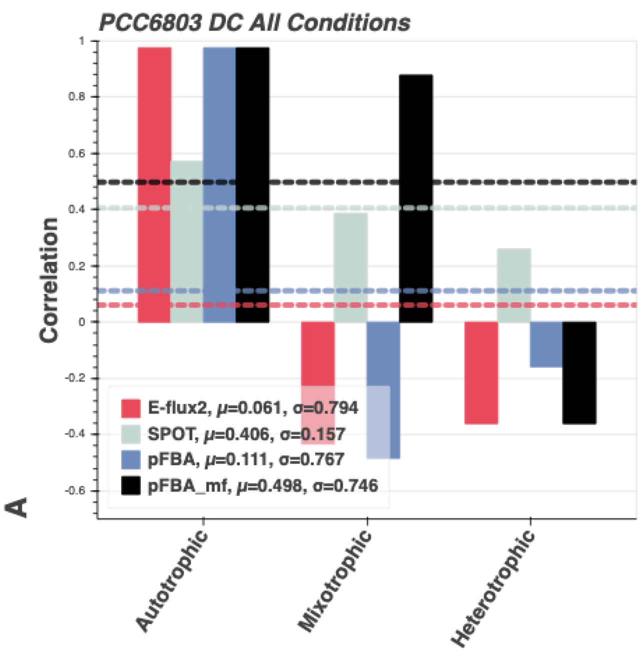

S2 Fig. PCC 6803 pFBA_mf vs pFBA: Similar to Fig 3 in the main text but shows that provided measured uptake rates with pFBA_mf (black) did not significantly improve the autotrophic flux prediction compared to pFBA (blue).

A)

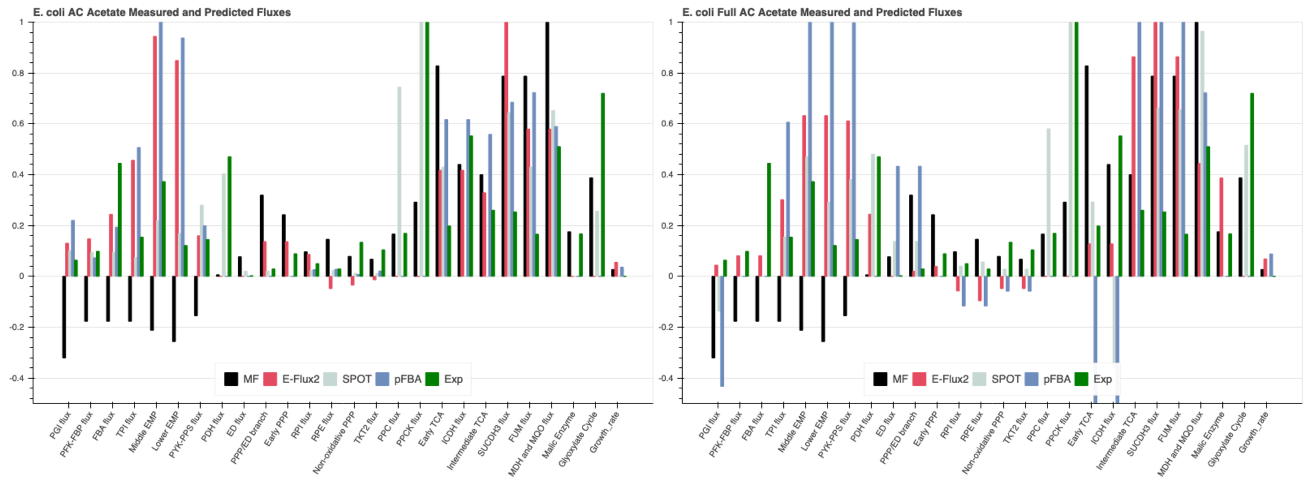
E. coli AC Acetate Measured and Predicted Fluxes
E. coli Full AC Acetate Measured and Predicted Fluxes
MF
E-Flux2
SPOT
pFBA
Exp
PGI flux
PFK-FBP flux
FBA flux
TPI flux
Middle EMP
Lower EMP
PYK-PPS flux
PDH flux
ED flux
PPP/ED branch
Early PPP
RPI flux
RPE flux
Non-oxidative PPP
TKT2 flux
PPC flux
PPCK flux
Early TCA
ICDH flux
Intermediate TCA
SUCDH3 flux
FUM flux
MDH and MQO flux
Malic Enzyme
Glyoxylate Cycle
Growth_rate

B)

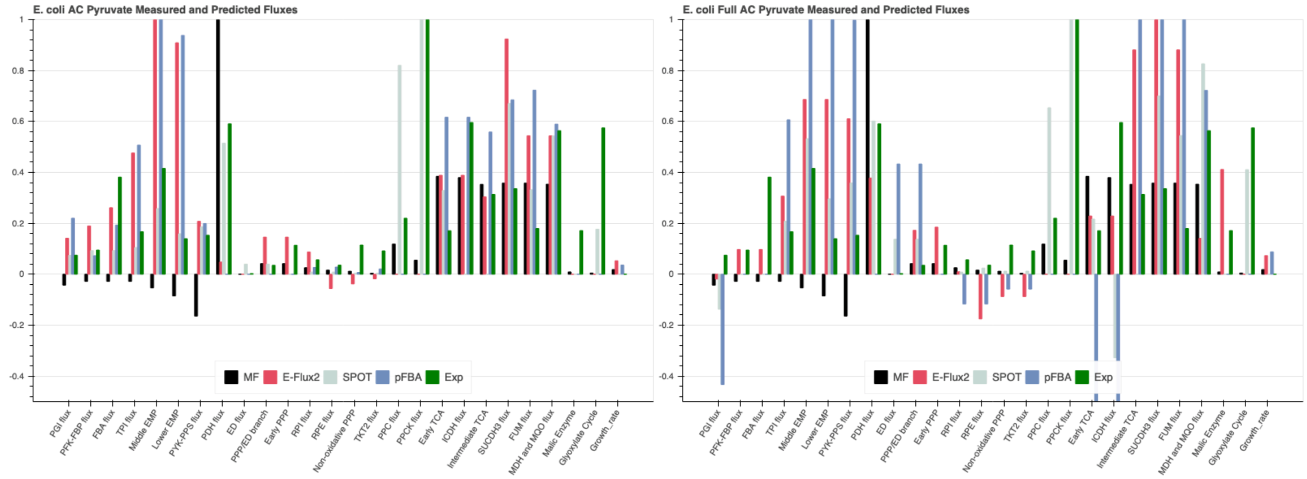
E. coli AC Pyruvate Measured and Predicted Fluxes
E. coli Full AC Pyruvate Measured and Predicted Fluxes
MF
E-Flux2
SPOT
pFBA
Exp

C)

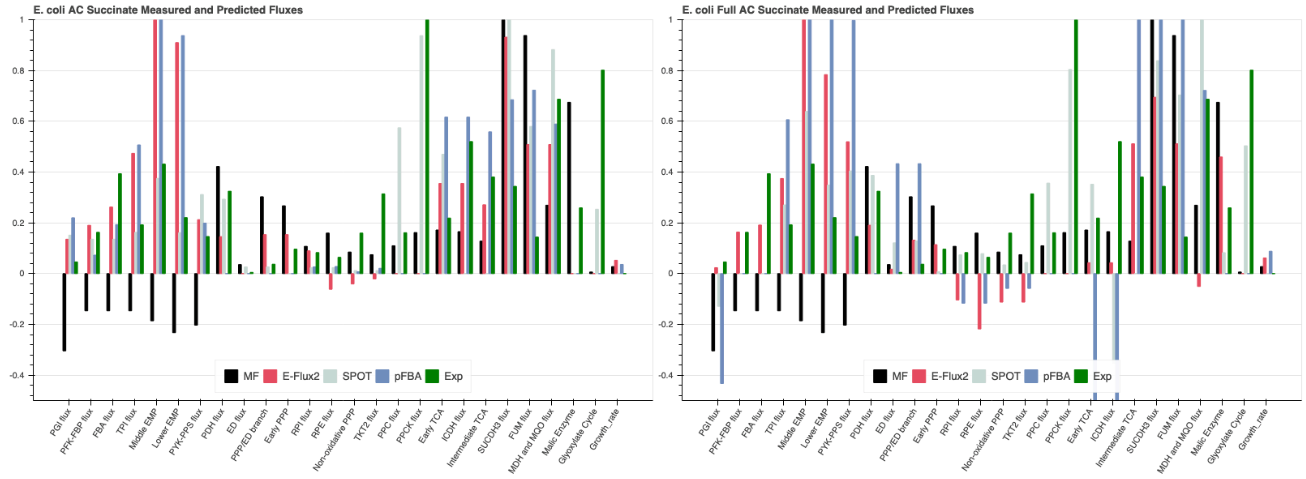
E. coli AC Succinate Measured and Predicted Fluxes
E. coli Full AC Succinate Measured and Predicted Fluxes
MF
E-Flux2
SPOT
pFBA
Exp

S3 Fig. *E. coli* flux directionality AC vs Full AC: Measured flux (black), E-Flux2 (red), SPOT (gray), pFBA (blue), and gene expression transcript abundance (green); all self-normalized to maximum values 1 and -1. Positive values reflect forward flux and negative values reflection reverse direction flux. The x-axis shows the measured central carbon flux reaction names. **(A)** Acetate data. **(B)** Pyruvate data. **(C)** Succinate data. These carbon sources had the lowest correlation values and these single carbon sources are also found in the TCA cycle. The difference in directionality between the black bars and the prediction bars highlights that glycolysis (PGI flux to PYK-PPS flux) and TCA cycle (RPI flux to TK2 flux) are being predicted in the opposite direction from the measured flux.

E. coli AC Acetate Measured and Predicted Fluxes

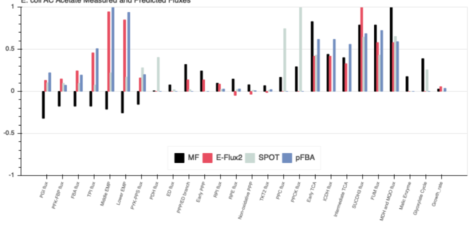

E. coli Full AC Acetate Measured and Predicted Fluxes

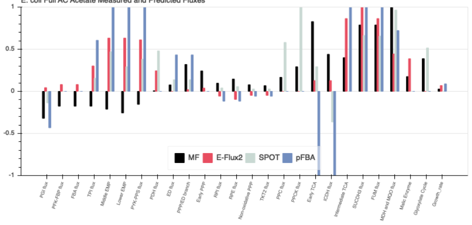

E. coli AC Fructose Measured and Predicted Fluxes

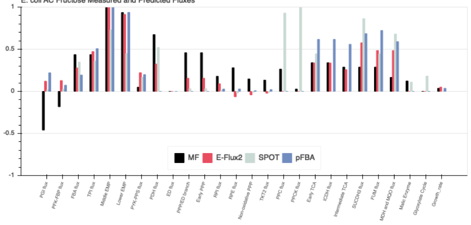

E. coli Full AC Fructose Measured and Predicted Fluxes

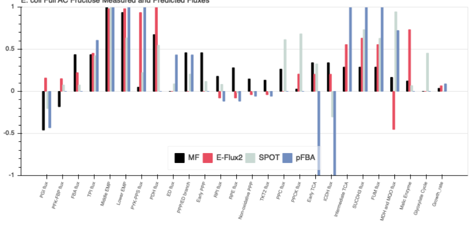

E. coli AC Galactose Measured and Predicted Fluxes

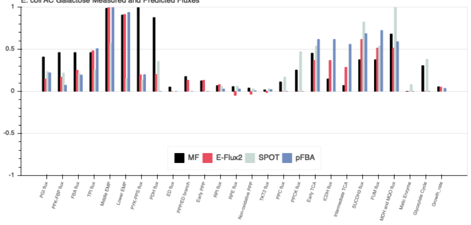

E. coli Full AC Galactose Measured and Predicted Fluxes

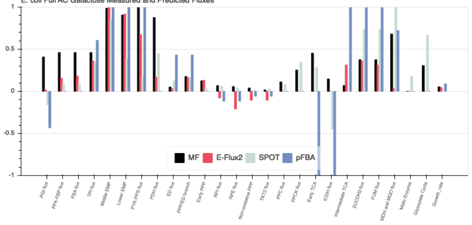

E. coli AC Gluconate Measured and Predicted Fluxes

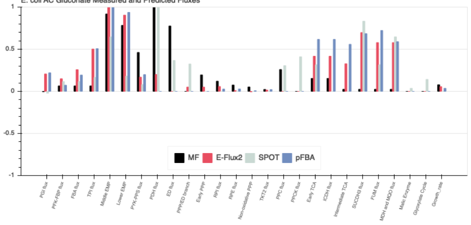

E. coli Full AC Gluconate Measured and Predicted Fluxes

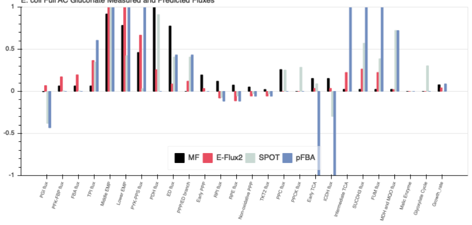

E. coli AC Glucose Measured and Predicted Fluxes

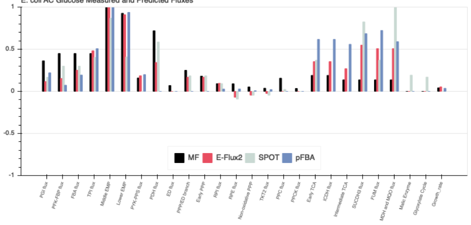

E. coli Full AC Glucose Measured and Predicted Fluxes

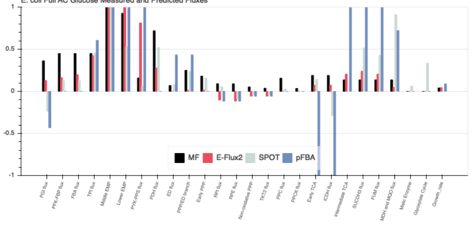

E. coli AC Glycerol Measured and Predicted Fluxes

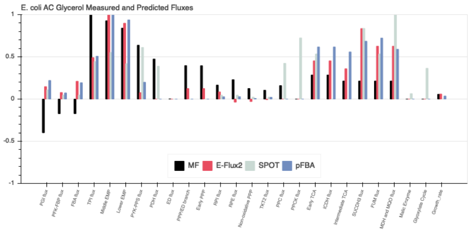

E. coli Full AC Glycerol Measured and Predicted Fluxes

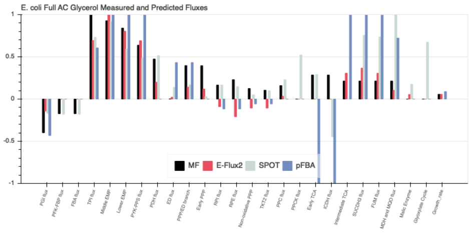

E. coli AC Pyruvate Measured and Predicted Fluxes

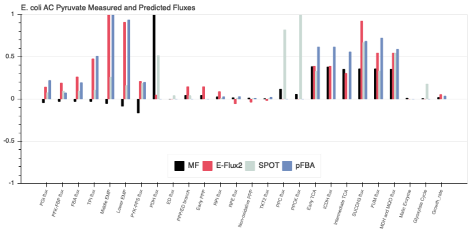

E. coli Full AC Pyruvate Measured and Predicted Fluxes

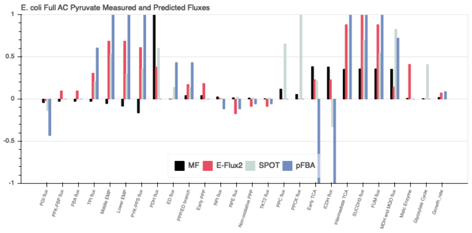

E. coli AC Succinate Measured and Predicted Fluxes

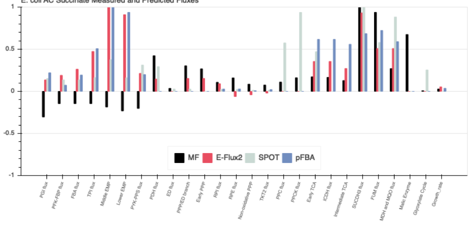

E. coli Full AC Succinate Measured and Predicted Fluxes

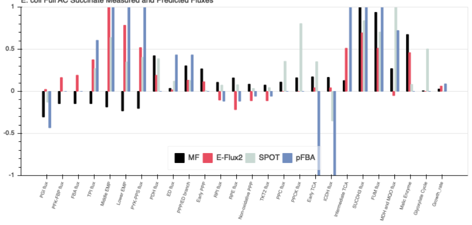

S4 Fig. *E. coli* flux directionality AC vs Full AC all carbon sources: Measured flux (black), E-Flux2 (red), SPOT (gray), pFBA (blue), and gene expression transcript abundance (green); all self-normalized to maximum values 1 and -1. Positive values reflect forward flux and negative values reflection reverse direction flux. The x-axis shows the measured central carbon flux reaction names.

B. sub AC Fructose Measured and Predicted Fluxes

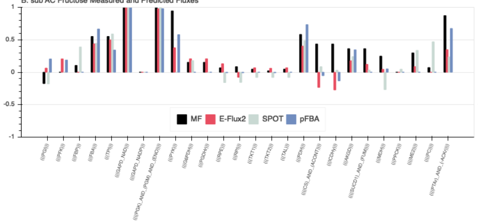

B. sub Full AC Fructose Measured and Predicted Fluxes

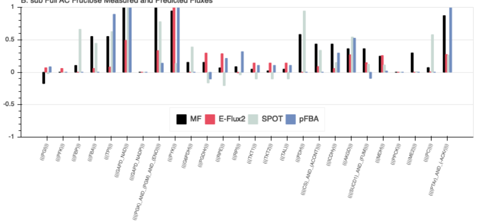

B. sub AC Gluconate Measured and Predicted Fluxes

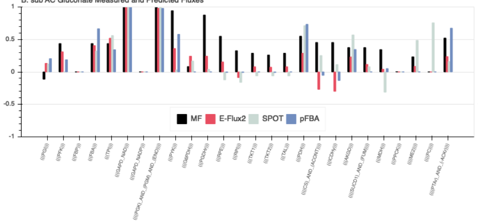

B. sub Full AC Gluconate Measured and Predicted Fluxes

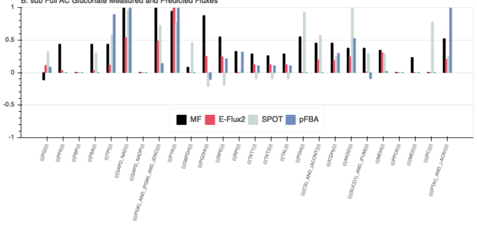

B. sub AC Glucose Measured and Predicted Fluxes

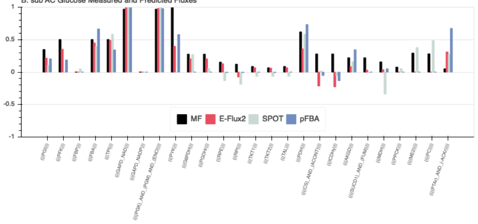

B. sub Full AC Glucose Measured and Predicted Fluxes

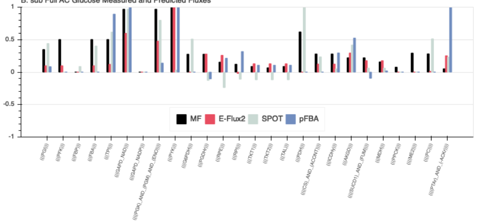

B. sub AC Glutamate+Succinate Measured and Predicted Fluxes

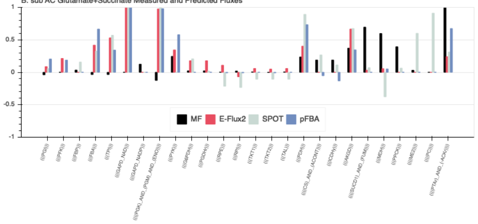

B. sub Full AC Glutamate+Succinate Measured and Predicted Fluxes

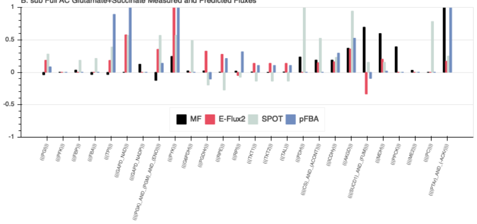

B. sub AC Glycerol Measured and Predicted Fluxes

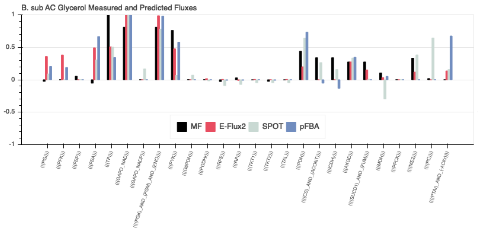

B. sub Full AC Glycerol Measured and Predicted Fluxes

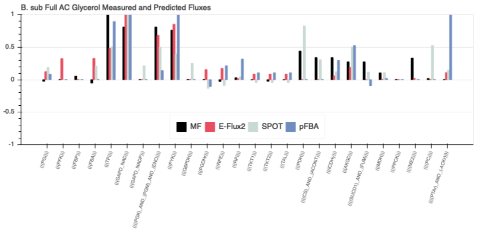

B. sub AC Malate Measured and Predicted Fluxes

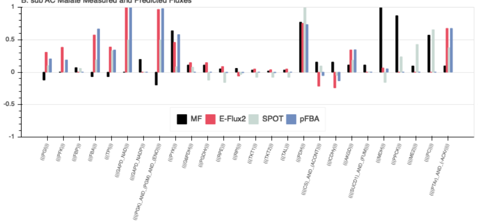

B. sub Full AC Malate Measured and Predicted Fluxes

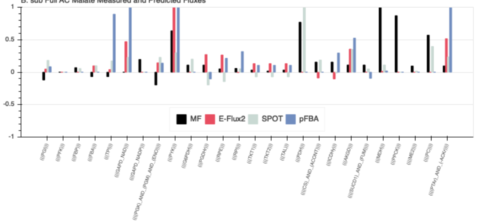

B. sub AC Mal+Glucose Measured and Predicted Fluxes

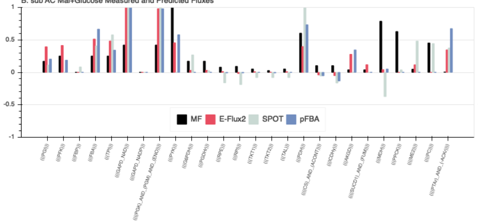

B. sub Full AC Mal+Glucose Measured and Predicted Fluxes

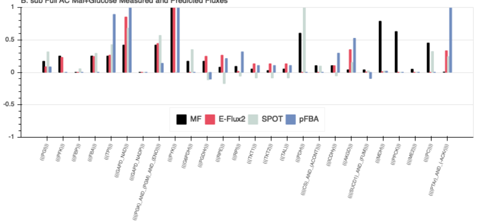

B. sub AC Pyruvate Measured and Predicted Fluxes

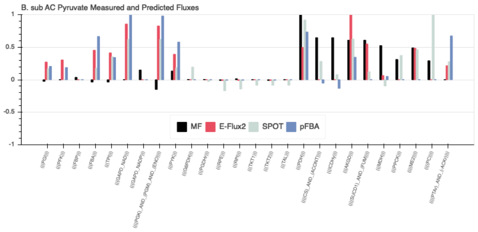

B. sub Full AC Pyruvate Measured and Predicted Fluxes

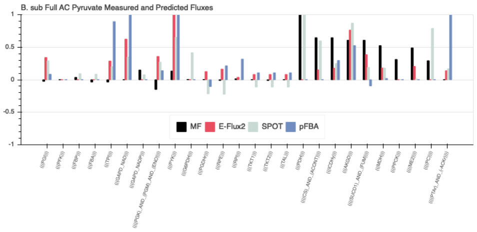

S5 Fig. *B. subtilis* flux directionality AC vs Full AC all carbon sources: Measured flux (black), E-Flux2 (red), SPOT (gray), pFBA (blue), and gene expression transcript abundance (green); all self-normalized to maximum values 1 and -1. Positive values reflect forward flux and negative values reflection reverse direction flux. The x-axis shows the measured central carbon flux reaction names.

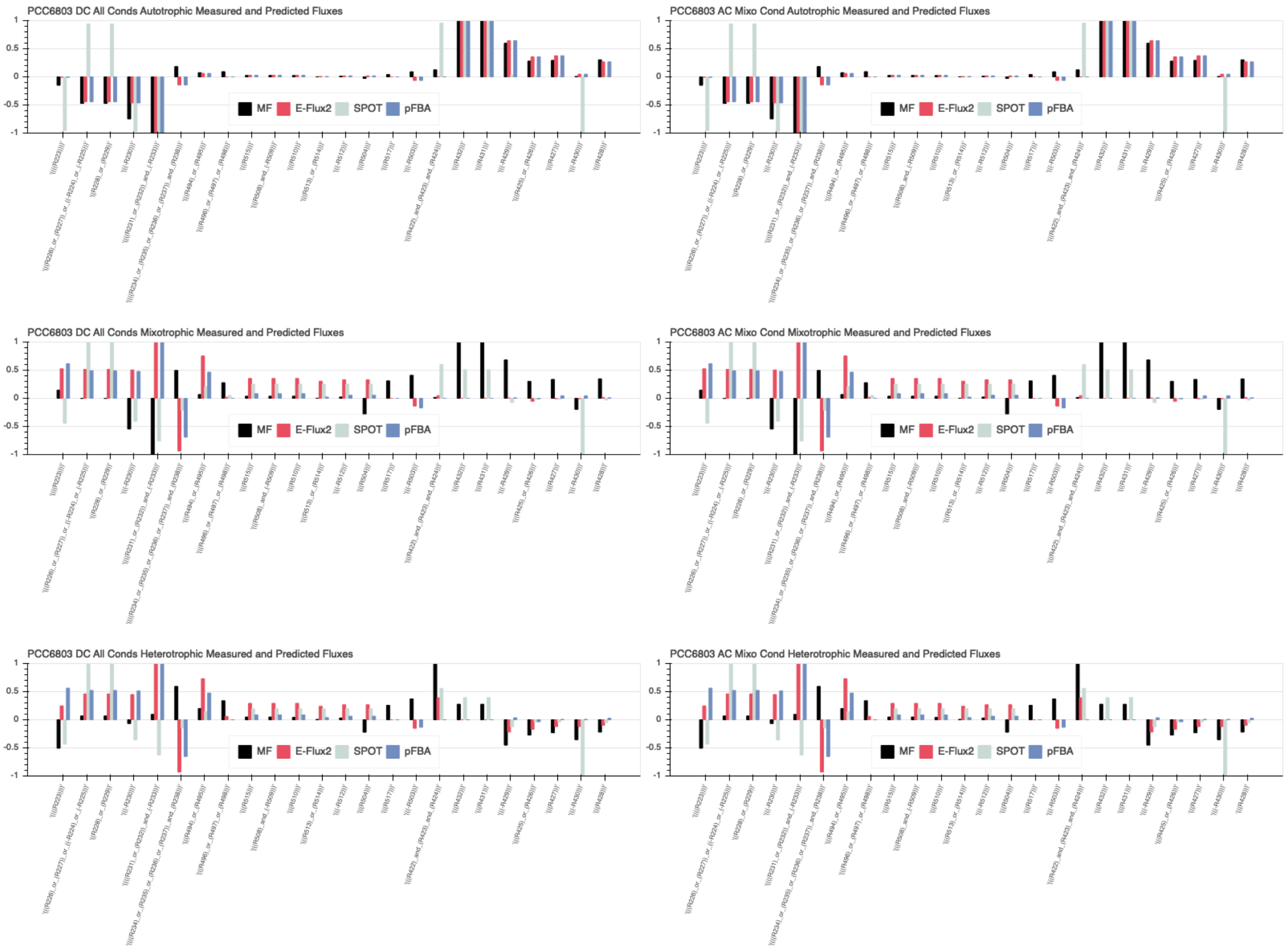

S6 Fig PCC 6803 flux directionality DC and AC conditions: Measured flux (black), E-Flux2 (red), SPOT (gray), pFBA (blue), and gene expression transcript abundance (green); all self-normalized to maximum values 1 and -1. Positive values reflect forward flux and negative values reflection reverse direction flux. The x-axis shows the measured central carbon flux reaction names.

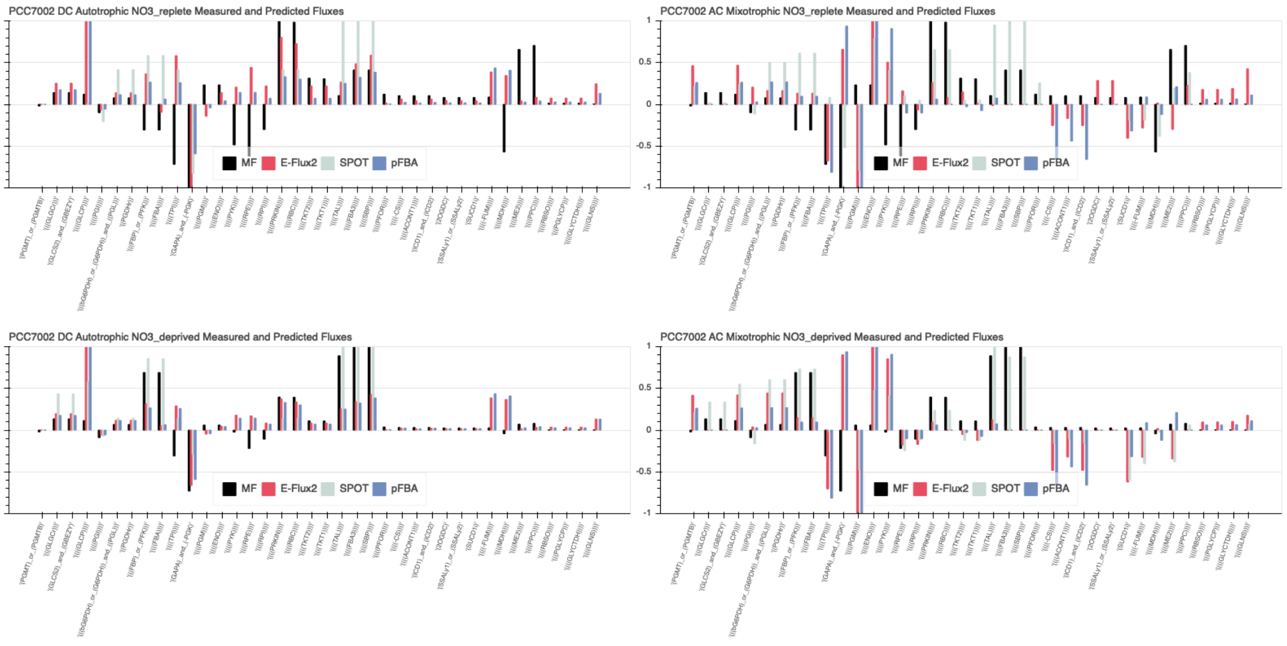

S7 PCC 7002 flux directionality DC and AC conditions: Measured flux (black), E-Flux2 (red), SPOT (gray), pFBA (blue), and gene expression transcript abundance (green); all self-normalized to maximum values 1 and -1. Positive values reflect forward flux and negative values reflection reverse direction flux. The x-axis shows the measured central carbon flux reaction names.

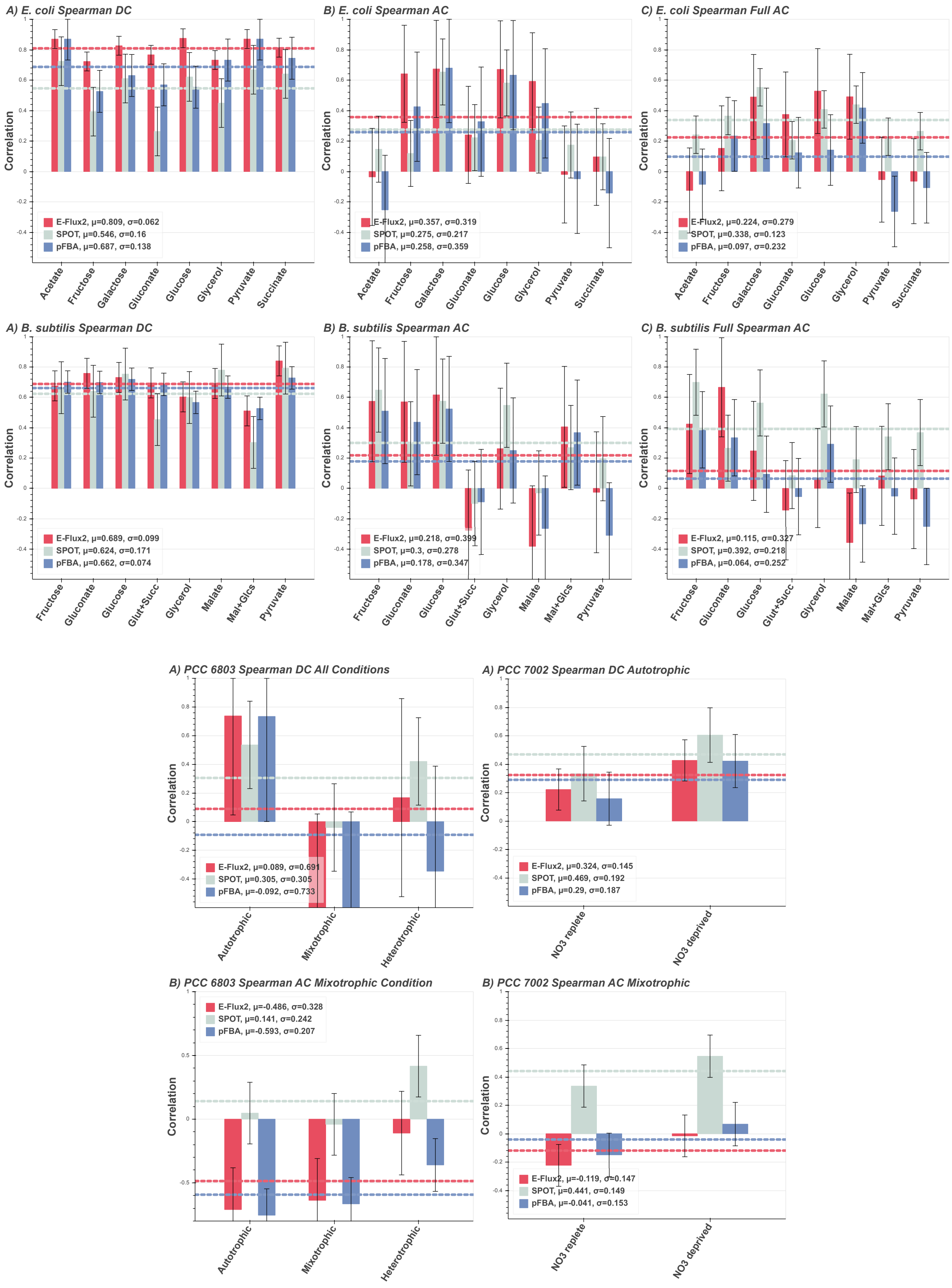
A) E. coli Spearman DC
E-Flux2, μ=0.809, σ=0.062
SPOT, μ=0.546, σ=0.16
pFBA, μ=0.687, σ=0.138
B) E. coli Spearman AC
E-Flux2, μ=0.357, σ=0.319
SPOT, μ=0.275, σ=0.217
pFBA, μ=0.258, σ=0.359
C) E. coli Spearman Full AC
E-Flux2, μ=0.224, σ=0.279
SPOT, μ=0.338, σ=0.123
pFBA, μ=0.097, σ=0.232
Acetate
Fructose
Galactose
Gluconate
Glucose
Glycerol
Pyruvate
Succinate
A) B. subtilis Spearman DC
E-Flux2, μ=0.689, σ=0.099
SPOT, μ=0.624, σ=0.171
pFBA, μ=0.662, σ=0.074
B) B. subtilis Spearman AC
E-Flux2, μ=0.218, σ=0.399
SPOT, μ=0.3, σ=0.278
pFBA, μ=0.178, σ=0.347
C) B. subtilis Full Spearman AC
E-Flux2, μ=0.115, σ=0.327
SPOT, μ=0.392, σ=0.218
pFBA, μ=0.064, σ=0.252
Fructose
Gluconate
Glucose
Glut+Succ
Glycerol
Malate
Mal+Glcs
Pyruvate
A) PCC 6803 Spearman DC All Conditions
E-Flux2, μ=0.089, σ=0.691
SPOT, μ=0.305, σ=0.305
pFBA, μ=-0.092, σ=0.733
A) PCC 7002 Spearman DC Autotrophic
E-Flux2, μ=0.324, σ=0.145
SPOT, μ=0.469, σ=0.192
pFBA, μ=0.29, σ=0.187
B) PCC 6803 Spearman AC Mixotrophic Condition
E-Flux2, μ=-0.486, σ=0.328
SPOT, μ=0.141, σ=0.242
pFBA, μ=-0.593, σ=0.207
Autotrophic
Mixotrophic
Heterotrophic
B) PCC 7002 Spearman AC Mixotrophic
E-Flux2, μ=-0.119, σ=0.147
SPOT, μ=0.441, σ=0.149
pFBA, μ=-0.041, σ=0.153
NO3 replete
NO3 deprived
Correlation

S8 Spearman correlations equivalents of main figures 1-4: Figures 1 through 4 from the main paper have been recreated using another correlation calculation, the Spearman rank correlation. The correlations are similar to the Pearson correlation from the main paper but a shift of lower correlation between measured vs predicted flux.

E. coli								
**Physiology**	**Acetate**	**Fructose**	**Galactose**	**Glucose**	**Glycerol**	**Gluconate**	**Pyruvate**	**Succinate**
Growth rate (h^{-1})	0.29 ± 0.01	0.49 ± 0.01	0.18 ± 0.01	0.65 ± 0.01	0.49 ± 0.01	0.59 ± 0.01	0.39 ± 0.01	0.51 ± 0.01
Uptake rate carbon source (mmol gCDW-1 h-1)	13.58 ± 0.33	8.33 ± 0.29	1.97 ± 0.10	9.65 ± 0.04	10.14 ± 0.15	7.28 ± 0.03	26.71 ± 0.79	15.90 ± 0.31
Acetate secretion (mmol gCDW-1 h-1)	-	3.33 ± 0.33	-	6.83 ± 1.03	0.60 ± 0.20	5.00 ± 0.16	11.91 ± 0.53	3.32 ± 0.31
Lactate secretion (mmol gCDW-1 h-1)	-	-	-	-	-	-	1.16 ± 0.07	-
Fumarate secretion (mmol $gCDW^{-1}$ h^{-1})	-	-	-	-	-	-	-	1.14 ± 0.07
Biomass yield (g g-1)	0.35 ± 0.00	0.33 ± 0.01	0.46 ± 0.03	0.37 ± 0.00	0.47 ± 0.01	0.41 ± 0	0.16 ± 0.01	0.26 ± 0.01

B. subtilis								
**Specific Rate**	**(mmol g-1 h-1)**	**(mmol g-1 h-1)**	**(mmol g-1 h-1)**	**(mmol g-1 h-1)**	**(mmol g-1 h-1)**	**(mmol g-1 h-1)**	**(mmol g-1 h-1)**	**(mmol g-1 h-1)**
**glucose**	7.36	-	-	-	-	-	5.95	-
**Fructose**	-	5.72	-	-	-	-	-	-
**gluconate**	-	-	5.13	-	-	-	-	-
**Succinate**	-	-	-	3.35	-	-	-	-
**glutamate**	-	-	-	2.21	-	-	-	-
**Glycerol**	-	-	-	-	6.22	-	-	-
**malate**	-	-	-	-	-	26.51	14.6	-
**pyruvate**	-	-	-	-	-	-	-	8.26

**PCC 6803**			
**Specific Rate**	**Autotroph mmol/g DCW/h**	**Mixotroph mmol/g DCW/h**	**Heterotroph mmol/g DCW/h**
**glucose**	0	0.24	0.41
**CO2**	0	1000	1000
**HCO3**	3.7	0	0
**Photons**	1000	1000	50

**PCC 7002**		
**Specific Rate**	**NO3 replete (mmol g-1 h-1)**	**NO3 depleted (mmol g-1 h-1)**
**CO2**	-29.5	-9.67
**NO3**	-1000	0

S1 Table. Measured uptake rates: Measured uptake rates for the four organism models (E. coli, B. subtilis, PCC 6803, and PCC7002), sourced from literature (See Materials and Methods 2.1). For *E. coli,* mean values and standard deviations

were obtained from 3 biological replicates. Any uptake rate set to 1000 is based on information indicating unconstrained uptake of the respective metabolite. In PCC 6803, the light inhibited culture was PSII chemically inhibited and cultured under aluminum foil. In an attempt to realistically constrain this experimental environment and chemical inhibition on the light reaction, 50 photon units were applied rather than 0. Additionally, this culture was grown in open air and therefore the CO_2 intake is left unconstrained. All uptake rates are present in units of mmol/g DCW/h.

Rxn#	Rxn Metabolites	E-Flux2	SPOT	pFBA
R419	Glycerone phosphate + D-Erythrose 4-phosphate --> Sedoheptulose 1,7-bisphosphate	0.0000	0.1922	0.0000
R420	Glycerone phosphate + D-Erythrose 4-phosphate --> Sedoheptulose 1,7-bisphosphate	0.0000	0.0000	0.0000
R421	H2O + Sedoheptulose 1,7-bisphosphate --> Orthophosphate + Sedoheptulose 7-phosphate	0.0000	0.1922	0.0000
R422	D-Glucose 6-phosphate + NADP+ --> D-Glucono-1,5-lactone 6-phosphate + NADPH + H+	0.0000	0.0225	0.0000
R423	D-Glucono-1,5-lactone 6-phosphate + H2O --> 6-Phospho-D-gluconate	0.0000	0.0225	0.0000
R424	6-Phospho-D-gluconate + NADP+ --> NADPH + H+ + CO2 + D-Ribulose 5-phosphate	0.0000	0.0225	0.0000
R425	D-Ribose 5-phosphate <==> D-Ribulose 5-phosphate	0.0030	0.0002	-0.0031
R426	D-Ribose 5-phosphate <==> D-Ribulose 5-phosphate	0.0030	0.0002	-0.0031
R427	D-Glyceraldehyde 3-phosphate + Sedoheptulose 7-phosphate <==> D-Xylulose 5-phosphate + D-Ribose 5-phosphate	0.0099	0.0004	0.0011
R428	D-Glyceraldehyde 3-phosphate + D-Fructose 6-phosphate <==> D-Xylulose 5-phosphate + D-Erythrose 4-phosphate	-0.0004	0.0004	0.0050
R429	D-Ribulose 5-phosphate <==> D-Xylulose 5-phosphate	0.0034	-0.0007	-0.0061
R430	D-Glyceraldehyde 3-phosphate + Sedoheptulose 7-phosphate <==> D-Fructose 6-phosphate + D-Erythrose 4-phosphate	-0.0099	0.1918	-0.0011

S2 Table. PCC 6803 Pentose Phosphate Pathway Fluxes: Predicted fluxes for 12 pentose phosphate pathway reactions for PCC 6803 under the heterotrophic growth condition and using heterotrophic gene expression data. Fluxes vectors were normalized by the l2 norm of the genome scale flux vector. Fluxes marked yellow show net flux values that are essentially zero flux (fluxes less than 1e-6). Green shows the net positive flux values. Red shows net negative flux values predicted, suggesting predicting the reversal of the pentose phosphate pathway. In SPOT, higher flux is passing through the pentose phosphate reactions, while in the E-Flux2 and pFBA show significantly less flux.
