# Supplementary material for: Assessment of transcriptomic constraint-based methods for central carbon flux inference
Source: PLoS One. 2020 Sep 9;15(9):e0238689. doi: 10.1371/journal.pone.0238689 (PMC7480874; doi:10.1371/journal.pone.0238689)
Supplement: Attachment — Submitted filename: Assessment of transcriptomic constraint-based methods for central carbon flux.pdf [file pone.0238689.s007.pdf]

Assessment of transcriptomic constraint-based methods for central carbon flux inference

Summary: In this paper, the authors sought to understand which methods (E-Flux2, SPOT or pFBA) best predicts intercellular flux when certain data types for experiment is missing (e.g. uptake rates, primary carbon usage etc). By systematically comparing the results of the methods with experimental C-13 MFA, they were able to determine the power of each method to correctly predict intracellular metabolic flux given the data constraints. Furthermore, they also analyzed data from non-model organisms of genus *Synechococcus* to ensure that their assessment were not model organism specific. Based on these thorough comparisons, the authors propose a decision tree that helps modellers choose the best method given the available data (Figure 5).

While the authors were thorough in their data collection, they were not as thorough in their analysis. The paper can be greatly strengthened by presenting variance in correlations between the model prediction and the experimental result. Without this information, it is difficult to determine the significance of difference between the different methods. The paper could also be strengthened by benchmarking it against other algorithms for integrating transcriptomics data (e.g. GIMME and iMAT). Overall, the exhaustive data collected/ analyzed and the decision tree produced by the paper would be a very useful guide to modellers working with incomplete dataset.

Major Comments:

1. In cases of low correlations between predicted and experimentally measured fluxes, it would be interesting to know if any particular pathway or subsystem contributes disproportionately to low correlation. These may point to failure points in the model and direct future improvements. This is briefly addressed on Line 172, but an expanded analysis could strengthen the paper.
2. Linde 177-178: “In speculative carbon sources, Fig 2B, all three methods perform similarly on average for AC. pFBA performs the best for the double carbon cases..”
    a. These conclusions are difficult to make for these conditions. pFBA’s performance on double carbon sources for AC is only slightly better (if at all). On ‘mal + glcs’ pFBA is on par with E-Flux2.
3. Figure 3B: Though the correlations are negative, there is a strong correlation between the pFBA flux prediction and the measured flux prediction (r = ~0.64). It is not clear from the text why the correlation is so strong and what this implies about the pFBA prediction in this sample/condition.
4. The crux of the paper relies on comparing correlations between experimental and simulation data. However, almost all correlations are presented as the mean with little information about variance. This makes it difficult to understand how significant the differences in correlations between the different methods are (see #3 above). We suggest the authors calculate total variance in correlation.
5. The authors should consider adding analysis with GIMME and iMAT. We understand the authors have previously done similar comparisons between GIMME, iMAT and E-Flux2

+ SPOT (Kim et al. 2016). However, with the addition of new organisms in this paper, it would be of interest to know how these different methods perform relative to each other. If E-Flux2 and SPOT still outperform GIMME and iMAT in these new conditions, it may also lead to greater usage of their methods in the future.

Minor Comments:

1. If there are future rounds of reviews, please provide higher quality figures (specially figures 1 and 2). Currently they are difficult to read.
2. Line 165: Change “supplied with 8 the carbon sources” to “supplied with all 8 carbon sources.”
3. Figure 1B: The actual results should be described in the text beyond just description of what the analysis was (e.g. “Fluxes during glucose showed the highest correlation with….”).
