# Supplementary material for: Assessment of transcriptomic constraint-based methods for central carbon flux inference
Source: PLoS One. 2020 Sep 9;15(9):e0238689. doi: 10.1371/journal.pone.0238689 (PMC7480874; doi:10.1371/journal.pone.0238689)
Supplement: Attachment — Submitted filename: Sid_PLosONE_rebuttal1_final.docx [file pone.0238689.s008.docx]

Tuesday, Dec 10, 2019

Dr. Christopher Boddy

Academic Editor, PLOS ONE

**RE: Manuscript ID: PONE-D-19-22421**

Title: Assessment of transcriptomic constraint-based methods for central carbon flux inference

Dear Dr. Boddy,

Thank you for considering the above-mentioned manuscript for publication. The additional comments and suggestions from the reviewers were very helpful. We have addressed those comments in the manuscript and provided the point-by-point answers below.

We are also including the revised manuscript for further consideration. I hope that you find the changes acceptable and complete. This manuscript is not being considered for publication elsewhere.

Thank you for your time and consideration.

Sincerely,

Desmond Lun

Professor
Department of Computer Science, Rutgers University–Camden

Department of Plant Biology, Rutgers University–New Brunswick

Tel.: +1-856-225-6094

E-mail: dslun@rutgers.edu

**Reviewer #1:** In this paper, the authors sought to understand which methods (E-Flux2, SPOT or pFBA) best predicts intercellular flux when certain data types for experiment is missing (e.g. uptake rates, primary carbon usage etc). By systematically comparing the results of the methods with experimental C-13 MFA, they were able to determine the power of each method to correctly predict intracellular metabolic flux given the data constraints. Furthermore, they also analyzed data from non-model organisms of genus Synechococcus to ensure that their assessment were not model organism specific. Based on these thorough comparisons, the authors propose a decision tree that helps modellers choose the best method given the available data (Figure 5).	While the authors were thorough in their data collection, they were not as thorough in their analysis. The paper can be greatly strengthened by presenting variance in correlations between the model prediction and the experimental result. Without this information, it is difficult to determine the significance of difference between the different methods. The paper could also be strengthened by benchmarking it against other algorithms for integrating transcriptomics data (e.g. GIMME and iMAT). Overall, the exhaustive data collected/ analyzed and the decision tree produced by the paper would be a very useful guide to modellers working with incomplete dataset.

Response**:** Thank you Reviewer #1 for your insightful comments. They were very helpful in improving the manuscript.

**Major comments:**

In cases of low correlations between predicted and experimentally measured fluxes, it would be interesting to know if any particular pathway or subsystem contributes disproportionately to low correlation. These may point to failure points in the model and direct future improvements. This is briefly addressed on Line 172, but an expanded analysis could strengthen the paper.

Response**:** Subsystem and low correlations analysis has been helpful in strengthening the manuscript.

We now expand upon cases where there are low correlations for all 3 methods, which suggests something more nuanced is happening under the blanket correlation value, and method based differences.
To highlight cases where all three methods showed poor correlation in the different carbon sources, supplementary figures S3 A, B, and C for *B. subtilis* have been replaced with *E. coli* data for the TCA cycle carbon sources (Acetate, Pyruvate, and Succinate) for which all 3 methods performed similarly low. The measured flux bars (black) are pointing in different directions than the predicted bars (colored by method), particularly for the initial glycolysis reactions and for the TCA reactions.

For expanded analysis we have included figures similar to Figure S3 for all organisms and condition, but without gene expression bars. (Fig S4 – S7). These figures show normalized measured flux vs. predicted fluxes for the 3 methods, in AC and Full AC carbon availability cases, as well as the growth condition cases. These figures support that, in cases of low correlation, the measured flux values for subset pathways were predicted in the opposite direction.

For future improvement, directionality prediction may be addressed. The initial reaction in a pathway effects the flux direction in the subsequent pathway. Therefore, predicting directionality incorrectly can lead to much lower correlation predictions.

Added Line 196 -202: “All three methods produce lower correlations for carbon sources found in the TCA cycle (**Fig 1C** Full AC acetate, pyruvate, and succinate). These low correlations were investigated and determined to be due to predicting flux opposite in direction to the measured flux (**Fig S3**). The measured fluxes for glycolysis are negative in reaction direction and the predictions are positive, while the measured fluxes for TCA cycle reactions are positive, and the predicted fluxes are negative. SPOT maintains higher correlations compared to E-Flux2 and pFBA due to predicting the TCA cycle reactions in the correct direction.”

Added Line 355 – 356: “For future improvement, developing a method for better determining flux directionality based on gene expression values should improve flux transcriptomic flux prediction.”

Line 177-178: “In speculative carbon sources, Fig 2B, all three methods perform similarly on average for AC. pFBA performs the best for the double carbon cases...” These conclusions are difficult to make for these conditions. pFBA’s performance on double carbon sources for AC is only slightly better (if at all). On ‘mal + glcs’ pFBA is on par with E-Flux2.

Response**:** We agree with the reviewer. pFBA and E-Flux2 preformed very similarly for

‘mal+glcs’ in *B. subtilis* AC.

We have replaced the above with this conclusion:

Line 209-210: “pFBA performs similarly poorly to the other methods for the double carbon cases and only marginally better for glutamate + succinate (see Discussion).”

Figure 3B: Though the correlations are negative, there is a strong correlation between the pFBA flux prediction and the measured flux prediction (r = ~0.64). It is not clear from the text why the correlation is so strong and what this implies about the pFBA prediction in this sample/condition. The crux of the paper relies on comparing correlations between experimental and simulation data. However, almost all correlations are presented as the mean with little information about variance. This makes it difficult to understand how significant the differences in correlations between the different methods are (see #3 above). We suggest the authors calculate total variance in correlation.

Response**:** The largest factor influencing the measured vs predicted flux correlation is the directionality of the predicted flux. The added supplementary figures (S3 – S7) support this conclusion. Strong correlations show matching directionality between predicted and measured fluxes, while poor correlations have non-matching directionality. A strong negative correlation is shown by predicting the opposite direction flux to the measure flux.

Variability has been included to all bar plots in the form or error bars, showing a spread of one standard deviation above and below the correlation value.

The authors should consider adding analysis with GIMME and iMAT. We understand the authors have previously done similar comparisons between GIMME, iMAT and E-Flux2 + SPOT (Kim et al. 2016). However, with the addition of new organisms in this paper, it would be of interest to know how these different methods perform relative to each other. If E-Flux2 and SPOT still outperform GIMME and iMAT in these new conditions, it may also lead to greater usage of their methods in the future.

Response**:** While seeing the performance of GIMME, iMAT, and other transcriptomic methods in addition to E-Flux2 and SPOT would indeed be interesting, this paper is not so much about comparing transcriptomic methods among each other as it is about comparing transcriptomic and non-transcriptomic methods. We have chosen E-Flux2 and SPOT as representatives of the transcriptomic methods because prior publications such as Kim et al. 2016 and Machado and Herrgard 2014 suggest that they are among the best of the transcriptomic methods. Similarly, we have chosen pFBA as a representative of the non-transcriptomic methods because prior publications suggest that it is among the best of the non-transcriptomic methods. We consider deeper exploration of the methods among these two classes to be beyond the scope of the paper.

Kim et al. did not actually compare against GIMME and iMAT in their paper. They cited that GIMME and iMAT had been compared with E-Flux and with the Lee method, and the latter two had performed favorably in comparison.

Added to Introduction Lines 70-72: “E-Flux2 and SPOT were chosen as representative transcriptomic methods, and similarly pFBA was chosen as the representative non-transcriptomic method, because prior publications suggest they are among the best in their respective method classes [3, 4].”

**Reviewer #1:**

**Minor comments:**

If there are future rounds of reviews, please provide higher quality figures (specially figures 1 and 2). Currently they are difficult to read.

Response**:** The figures have been reproduced in much higher quality, using .svg format and then converted to .eps, as per the journal’s submission formatting.

Line 165: Change “supplied with 8 the carbon sources” to “supplied with all 8 carbon sources.”

Response**:** Line has been changed to “supplied with all 8 carbon sources.”

Figure 1B: The actual results should be described in the text beyond just description of what the analysis was (e.g. “Fluxes during glucose showed the highest correlation with….”).

Response**:** The legends for Fig 1 and 2 have been updated.

**Revised Fig 1 (*E. coli*) legend:** Correlations between measured and predicted flux of *E. coli* grown on 8 different carbon sources for the three FBA methods E-Flux2 (red), SPOT (gray), pFBA (blue). Horizontal dashed lines are the respectively colored mean correlations per method. The mean (µ) is the average prediction correlation per method. The standard deviation (σ) is the spread of prediction correlation above and below the mean, denoted by the error bars. **(A)** Respective direct carbon source (DC) supplied. pFBA was given the additional constraint of known uptake rate in the single carbon source, while E-Flux2 and SPOT were not. All methods perform consistently across the individual carbon sources. **(B)** All 8 carbon sources supplied and correlated with measured flux from single carbon growth (AC). Correlations drop in all methods, particularly in the TCA cycle carbon sources (Acetate, Pyruvate, and Succinate). **(C)** All possible carbon sources in the model supplied (Full AC). All methods again lose performance, but the transcriptomic methods retain decent correlations. See Supplementary S1 Table for uptake rates used.

**Revised Fig 2 (*B. subtilis*) legend:** Correlations between measured and predicted flux of heterotrophic *B. subtilis* grown on 8 different carbon sources for the three FBA methods E-Flux2 (red), SPOT (gray), pFBA (blue). Double carbon sources are denoted as glutamate with succinate (Glut + Succ) and malate with glucose (Mal + Glcs). Horizontal dashed lines are the respectively colored mean correlations per method. The mean (µ) is the average prediction correlation per method. The standard deviation (σ) is the spread of prediction correlation above and below the mean, denoted by the error bars. **(A)** Respective direct carbon source (DC) supplied. pFBA was given the additional constraint of known uptake rate in the single carbon source, while E-Flux2 and SPOT were not. All methods perform consistently across the individual carbon sources, with minor drops in correlation for double carbon sources (Glut + Succ and Mal + Glcs). **(B)** All 8 carbon sources supplied and correlated with measured flux from single carbon growth (AC). Correlations drop in all methods, particularly for Malate. **(C)** All possible carbon sources in the model supplied (Full AC). All methods again lose performance, but the transcriptomic methods retain decent correlations. See Supplementary **S1 Table** for uptake rates used.

**Reviewer #2:**

Response: Thank you Reviewer #2 for your insightful comments. They were very helpful in improving the manuscript.

**Major comments:**

1) I would have liked to see a more extensive discussion of the experimental error involved in measuring fluxes via Carbon-13; for instance, what is known about the repeatability of these measurements?

Response**:** Thank you for pointing this out. Experimental error in 13C-MFA strengthens the conclusion that transcriptomic data in flux prediction is useful, as errors in 13C-MFA reduces the incentive to perform the experiment.

Added to the Introduction, Lines 77-79: Additionally, many published ^13^C-MFA studies are unable to be reproduced due to missing information, with “only 30% of the studies examined were found to be acceptable” in a review by Crown et al [15].

2) The correlation measure is very crude and hides a lot of nuances; for instance, not all fluxes are equally important to get right (the growth rate is typically the key quantity in model analysis, but also larger-scale pathway utilization may be of interest). Furthermore, it is very possible to have a perfect correlation, but be off by a factor of 10 in every single prediction - thus, not only should the correlation coefficient be reported, but also the equation of the line of best fit. An additional question could be about the similarities of the rankings between fluxes (e.g. Spearman correlation) because sometimes it is preferable to determine the relative importance of the reactions or pathways, rather than their absolute importance, to the cell’s internal state. In addition, the use of the correlation as the main metric is not ideal because the typical goal of a metabolic study is to obtain qualitative information about the internal state of the cell (often relative to a baseline), whereas the correlation tries to make a quantitative statement about it, which is often (though not always) an over-interpretation of the metabolic model’s predictions.

Response: We use correlation here because it is a good goodness-of-fit metric for this application. In general, transcriptomic flux inference methods produce fluxes in an arbitrary unit. This is because we estimate that a high number of transcripts corresponds to a high flux for a given reaction, but not what the underlying flux actually is. Therefore, having a perfect correlation while being off by a factor of 10 is a desirable feature of the metric; and not a bug.

To elucidate the contributing factors within each correlation, we have included additional supplementary figures (S3 – S7). These figures show normalized measured flux vs. predicted fluxes for the 3 methods, in AC and Full AC carbon availability cases, as well as the growth condition cases.

Spearman correlation figures were generated and included as well under supplementary figures (S8). We find that the Spearman correlations are similar to the Pearson correlations, though they are, in general, lower. Combined with the flux directionality figures (S3 – S7), it appears that the main contributor to the correlation values is due less to relative vs absolute magnitude but more to incorrect pathway directionality.

Added to Results and discussion Lines 146-149: “We have chosen the uncentered Pearson correlation as a goodness-of-fit metric because transcriptomic flux inference, in general, estimates that high transcript count corresponds with high flux, but not the actual flux value. Therefore, the predicted flux values are in arbitrary units. The uncentered Pearson correlation captures predictive accuracy irrespective of the scaling introduced by the expression data.”

3) It is unclear what the two growth conditions were used for PCC 7002 - only one of them is mentioned in the paper in the relevant section.

Response: The single growth condition used in 13C-MFA of PCC 7002 was photoautotrophic. A second artificial growth condition was used in flux prediction, which was constrained into the heterotrophic condition. This was done as an extension of our flux prediction method, where we attempted to predict flux using the second condition’s set of incorrect conditional constraints and see how gene expression might help reduce prediction error.

Our results show that SPOT still predicts flux even with incorrect conditional constraints set on a the PCC7002 autotrophic model.

Revised section in the Results, Extended Analysis: Artificial conditional information deficit:

Lines 319-327: “As an extension of our findings, PCC 7002 was constrained under a second artificial growth condition set to mimic mixotrophic conditions. We attempted to predict flux using the second condition’s set of incorrect conditional constraints and see how gene expression might help reduce prediction error. This allows for carbon sources other than CO_2_ allowed for uptake as well as unconstrained NO_3_ uptake for both the N replete and N deprived cases (**Fig 4B**). This mixotrophic state is not found in nature, and therefore the PCC 7002 central carbon flux distribution correlation was expected to be poor [32]. With the nitrate growth condition unspecified in the model, NO_3_ was allowed into the cell freely for both conditions. The correlations for E-Flux2 and pFBA were indeed poor, but SPOT produced strong correlations. This suggests that even in incorrectly constrained models supplied with unrealistic carbon sources and no secondary metabolite information, gene expression can still be used predict central carbon flux well (**S7 Fig**).

4) The figures (in particular, figures 1 and 2) are very grainy (low resolution) and do not use a consistent color scheme. They should be substantially improved. I recommend using ggplot2 in R for the plotting (alternatively, matplotlib in Python could work). You should also provide error bars on the plot, rather than having a mean and standard deviation specified in the legend.

Response: The figures have been reproduced in much higher quality, using .svg files and then converted to .eps, as per the journal’s submission formatting.

The color scheme has also been changed (black bars removed). The black bars were originally placed to show that pFBA was supplied the correct carbon source uptake rate, while E-Flux2 and SPOT were not. The black bar coloring has been removed from all figures, but the uptake rate information was still supplied. This is still mentioned in the manuscript and Figure S2 still has black bars as it conveys specific information about carbon source not affecting growth condition based predictions in autotrophic organisms.

Error bars were added to the figures as one standard deviation above and below the correlation value.

Figure 1 and 2 legends changed with: “pFBA was given the additional constraint of known uptake rate in the single carbon source, while E-Flux2 and SPOT were not.”

Line 188-189: “pFBA was provided an additional constraint to improve prediction with the experimentally measured carbon source uptake flux (uptake rate) being set within the pFBA runs only (**Fig 1A**).”

5) The organization of the Results and Discussion section needs to be improved - the information is there, but hard to digest in the way it is currently organized. I would recommend having the sections represent an organism and the subsections represent the setting, or vice versa; right now it is a complicated mixture of both, which prevents an easy overview of the results.

Response: We agree that the Results and Discussion organization is difficult to follow.

The Results and Discussion organization has been organized as Section as Setting then Subsection as Organism. To improve the flow this section, we have revised and added the following lines to match the section and subsection organization.

The Section and subsection headers and respective text have been renamed and moved. The new organization is as follows:

Results and discussion

Correlations for known and unknown carbon source

Central carbon flux correlations in E. coli

Central carbon flux correlations in B. subtilis

Discussion of known and unknown carbon source

Correlations for known and unknown growth condition

Central carbon flux correlations in PCC 6803

Central carbon flux correlations in PCC 7002

Discussion of known and unknown growth condition

Extended Analysis: Artificial conditional information deficit

Conclusion

Line 151-152: “Testing flux prediction under known and unknown carbon sources, *E. coli* and *B. subtilis* fluxes were simulated under different carbon source availabilities, at three different stages.”

Line 160-163: “Testing flux prediction under different growth condition in PCC 6803 and PCC 7002. Fluxes were simulated under based on the organism’s possible metabolic states, at two different stages.”

Line 186: “Under direct carbon source (DC) the *E.coli* models…”

6) I am not entirely convinced that the situation where the information about a cell’s growth conditions is incomplete is a realistic one. I would like to see the discussion at least one clear setting where that is likely to happen (or, barring that, an acknowledgment by the authors that the utility of the proposed approach is limited to a somewhat hypothetical scenario).

Response: Thank you for bringing this to our attention. We have added a more extensive discussion in the manuscript describing situations of incomplete growth condition information. We believe that, in practice, such situations are the ones of greater interest, as opposed to situations of growth in highly controlled lab conditions, where aside from the considerations of time and cost, ^13^C-MFA will be feasible and often preferable as a method of determining intracellular fluxes:

Added Lines 170-181 : “Unknown growth condition was used to demonstrate cases of complex media or *in vivo* growth of cultures. An example of this would be in studying the metabolism of enteric bacteria both pathogenic and commensal/mutualistic, in which the growth medium is complex, and the culture is grown *in vivo*. An example of a pathogenic model is *Mycobacterium tuberculosis*. In tuberculosis, the bacterium live inside of scar tissue of the lung and their metabolism is uncertain. To measure this using conventional ^13^C-MFA would not be feasible, but extraction of RNA expression data has been shown to be possible [28]. This may also be useful in other cases of bacterial pathogenesis. In the cases of commensalism/mutualism within the gut microbiome, the distribution of bacterial species in the gut has been shown to vary based on diet [29]. It may be possible to determine how metabolism in the microbes shift in the species that continue to persist in the gut during dietary changes, using transcriptomic flux prediction. A hypothetical experiment would be to sample RNA from the gut during a period of one type of host diet, then sample RNA again after a period of time on another diet. Although this is dependent on the expression profiles and metabolic models to be complete enough for prediction of central carbon metabolism.”

7) Can you learn something by combining the outputs of all three methods? For instance, is the average of the three predictions (or the two predictions by the methods using transcriptomics data) better correlated with the gold standard than the individual one? This could be an interesting finding in and of itself.

Response: The average of the three predictions (or the two transcriptomic methods) are not better correlated with the gold standard. A possible approach worth pursuing could be to combine the prediction results using a weighted average and then use machine learning to determine the weights**.** This is an interesting idea but beyond the scope of the current paper.

**Reviewer #2:** This paper addresses a limitation of the subfield of metabolic network analysis concerned with predicting flux distributions under specific growth conditions (specified by key nutrient sources and/or maximum uptake rates). It collates 21 settings that have both Cabon-13 flux measurements (considered to be a gold standard here) as well as transcriptomic measurements that can be used to constrain a metabolic model. The models and the transcriptomic data are then used to predict fluxes, and these are then compared to the gold standard. The authors find that transcriptomic-based methods can be advantageous over standard methods when the transcriptomic data is obtained in a setting with incomplete information about the cell’s growth conditions.

**Minor comments:**

Lines 43-44: Cells cultured on identical substrates produce highly conserved glucose metabolism pathways -> utilize highly similar pathways?

Response: Line changed to “utilize highly similar metabolic pathways”

Line 66: To this end, we have […] -> specify to what end?

Response: Line has been changed to “To address the limitations of the previous dataset,”

Line 83: Any log2 normalized data was exponentiated. -> I would say “any log-transformed data was transformed back to real scale by exponentiation” as normally, “exponentiated” suggests that the base of exponentiation was e, rather than 2.

Response: Line was changed to “Any log-transformed data was transformed back to their original scale by exponentiation.”

Line 89: We used iJO1366 as the genome-scale metabolic model. -> I would have liked to see a discussion of the effect of the model choice on the results, though I recognize that this might be outside of the scope of the current article.

Response: From the in-line reference [17], “*i*JO1366 is the most complete *E. coli* metabolic reconstruction to date” and “*i*JO1366 contains most of the best-characterized genes in *E. coli*” as of 2011. This may no longer be the case as there are other models, EcoCyc–18.0–GEM and BL21(DE3), that have more genes, but the iJO1366 model is more closely related to the strain BW25113, which was the strain used in the ^13^C-MFA *E. coli* experiments in our paper.

Orth JD, Conrad TM, Na J, Lerman JA, Nam H, Feist AM, et al. A comprehensive genome‐scale reconstruction of *Escherichia coli* metabolism—2011. Molecular Systems Biology. 2011;7(1):535.

Added to Results and discussion Line 144 – 145:“We expect model choice affects the transcriptional FBA methods more than the non-transcriptional. A less complete model may have reduced constraint mapping from the relevant gene expression data.”

Line 101: remove the extra “H” before the formula for bicarbonate

Response: Line has been changed.

Line 104: metabolites can exchange -> metabolites can be exchanged

Response: Line has been changed.

Line 116: All methods used in this study are referenced with their original publications -> from their original publications? The sentence is very confusing in any case.

Response: Line has been changed to “All FBA methods used in this study are referenced from their original publications.”

Line 118: When I follow the link I get the following error (please fix it): You don't have permission to access / on this server.

Response: The link, <http://most.ccib.rutgers.edu/> works now.

Line 139: see Methods 2.1 -> Section Methods 2.1

Response: This and all other occurrences of “see Methods #” have been changed to “Section Methods”

Line 310: incorrect hyphenation on both ends

Response: We do not see any incorrect hyphenation at this line.
