# Supplementary material for: Assessment of transcriptomic constraint-based methods for central carbon flux inference
Source: PLoS One. 2020 Sep 9;15(9):e0238689. doi: 10.1371/journal.pone.0238689 (PMC7480874; doi:10.1371/journal.pone.0238689)
Supplement: Attachment — Submitted filename: Sid_PLosONE_rebuttal2.docx [file pone.0238689.s009.docx]

Monday, July 6, 2020

Dr. Christopher Boddy

Academic Editor, PLOS ONE

**RE: Manuscript ID: PONE-D-19-22421**

Title: Assessment of transcriptomic constraint-based methods for central carbon flux inference

Dear Dr. Boddy,

Thank you for considering the above-mentioned manuscript for publication. We have addressed the minor revision comments in the manuscript and provided the point-by-point answers below.

We are also including the revised manuscript and hope that you find the changes acceptable and complete. This manuscript is not being considered for publication elsewhere.

Thank you for your time and consideration.

Sincerely,

Desmond Lun

Professor
Department of Computer Science, Rutgers University–Camden

Department of Plant Biology, Rutgers University–New Brunswick

Tel.: +1-856-225-6094

E-mail: dslun@rutgers.edu

**Reviewer #2:** I thank the authors for addressing my concerns; however, their modifications have introduced some additional concerns, as detailed below. I do not believe these to be a major obstacle to publication, however, and am therefore recommending minor revisions.

Response: The reviewer has made strong points and detailed comments. Thank you, again, for your time and bringing these to attention.

**Major comments:**

1) “enteric bacteria both pathogenic and commensal/mutualistic, in which the growth medium is complex,
and the culture is grown in vivo. An example of a pathogenic model is Mycobacterium tuberculosis.”

This incorrectly suggests that Mycobacterium tuberculosis is an enteric pathogen, which it is not (it is primarily, though not exclusively, found in the lungs)

Response: The line has been changed to, “enteric bacteria, in which the growth medium is complex, and the culture is grown *in vivo*. An example enteric model is *Mycobacterium tuberculosis*, …”

In tuberculosis, the bacterium live inside of scar tissue of the lung and their metabolism is uncertain.

Technically, the bacteria (plural!) live inside a granuloma, formed by macrophages, rather than “scar tissue”.

Response: “An example enteric model is *Mycobacterium tuberculosis*, in which using conventional ^13^C-MFA to measure latent bacteria would not be feasible due to additional constraints such as tissue specificity and slow *in vivo* growth rates, but extraction of RNA expression data has been shown to be possible.”

Also, please provide references; in particular, the work by Palsson’s group on modeling Mycobacterium tuberculosis inside a macrophage seems relevant to cite (other models may have been published since as well).

Response: Palsson’s group has been cited to support difficulty in Mycobacterium modeling.

Bordbar A, Lewis NE, Schellenberger J, Palsson BØ, Jamshidi N. Insight into human alveolar	macrophage and M. tuberculosis interactions via metabolic reconstructions. Molecular Systems	Biology. 2010;6(1):422.

“A hypothetical experiment would be to sample RNA from the gut during a period of one type of host diet, then sample RNA again after a period of time on another diet.”

Unfortunately, it is currently somewhat challenging to even determine the composition of a microbiome from RNA-Seq data at a particular timepoint with high accuracy; I think that expecting to detect changes in metabolism from this kind of data would be very ambitious (though some startups, such as Gusto, claim to be able to do that).

Response: In agreement, RNA-seq from the microbiome to detect metabolic changes is unlikely with current techniques. To better reflect this, the surrounding text and quoted text have been carefully rephrased such as “determine” has been changed to “detect” and “A hypothetical experiment…” has been changed to “A conjectured experiment…”

“With improved RNA extraction techniques, it may be possible to detect microbial metabolic shifts in species that continue to persist in the gut during dietary changes, using transcriptomic flux prediction. A conjectured experiment would be to sample RNA from the gut during a period of one type of host diet, then sample RNA again after a period of time on another diet. Although this is highly dependent on the quality of expression profiles and metabolic.”

**Grammar and minor comments:**

simulated under based -> simulated based

Response: Line has been changed.

Although this is dependent on the expression profiles and metabolic models to be complete enough for prediction of central carbon metabolism. - confusing!

Response: Line has been changed to “Although this is highly dependent on the quality of expression profiles and metabolic models.”

decent correlations -> reasonable correlations

Response: Line has been changed.

Double carbon sources are denoted as glutamate with succinate (Glut + Succ) and malate with glucose (Mal + Glcs). -> edit for grammar

Response: Line has been changed to “Double carbon sources are glutamate plus succinate (Glut + Succ) and malate plus glucose (Mal + Glcs).”

If carbon source is speculatively known, and uptake rate is unknown, as presented with a relatively small set of 8 possible carbon sources (AC), pFBA predictive power drops significantly -> same

Response: Line has been changed to “If carbon source is speculatively known, uptake rate is unknown, and presented with a relatively small set of 8 possible carbon sources (AC), pFBA predictive power drops significantly.”

where more closely resembles -> same

Response: Line has been changed “more closely resembles”

S3 – S5 Fig -> Fig. S3 – S5 Fig

Response: Line has been changed to “Fig. S3 – S5”

constrained under a second artificial growth condition was set to mimic -> edit for grammar

Response: Line has been changed to “constrained under a second artificial growth condition set to mimic mixotrophic conditions.”

be used predict -> same

Response: Line has been changed to “be used to predict”

flux transcriptomic flux prediction. -> repeated word

Response: Line has been changed to “transcriptomic flux prediction”

S1, S2, and S3, S4, S5, S6, S7, and S8 Figs -> same comment as above

Response: Line has been changed to (Figs. S1, S2, S3, S4, S5, S6, S7, and S8) and tables (Tables S1 and S2)

**Reviewer #3:** The authors present here the use of non-transcriptomic-based and transcriptomic-based methods to predict metabolic fluxes, where nutrient sources and uptake rates are available or not available. The authors show that non-transcriptomic-based and transcriptomic-based methods perform similarly with information available on carbon sources and uptake rates. However, transcriptomic-based methods are better at predicting flux distributions than constraint-based method, across various organisms and conditions.

The authors have sufficiently addressed the reviewer comments and the overall organization and structure of the manuscript has improved. I do not have further concerns except a few minor comments.

Response: Thank you for your time and comments, theses clarifications will help future readers.

**Minor comments:**

There is wording on nitrogen (or N) deprived throughout the manuscript. It should be nitrogen-deprived or N-deprived.

Response: First instances replaced as nitrogen-replete and nitrogen-deprived. All following instances replaced to N-replete and N-deprived, respectively.

The terms DC, AC and full AC appear before their full names were introduced.

Response: Line 152 has been edited to introduce the labels DC, AC, and Full AC along with their explanations.

Line 152: “…at three different stages labeled as AC, DC, and Full AC.

A. DC: Known carbon source and uptake rate information available, uptake rate is only supplied to the non-transcriptomic method, pFBA.
B. AC: Unknown carbon source and uptake rate, only eight speculative carbon sources without uptake rate data are available to the model.
C. Full AC: No carbon source information available, all possible carbon sources (and any other extracellular metabolites) opened for exchange into the model.”

Line 253 effects -> affects

Response: Line has been changed.
